# Gasotransmitters in Biology and Medicine: Molecular Mechanisms and Drug Targets

**DOI:** 10.1155/2016/4627308

**Published:** 2016-09-29

**Authors:** Guangdong Yang, Alp Sener, Yong Ji, Yanxi Pei, Michael D. Pluth

**Affiliations:** ^1^Department of Chemistry and Biochemistry, Laurentian University, Sudbury, ON, Canada; ^2^Department of Surgery and Department of Microbiology and Immunology, Western University, London, ON, Canada; ^3^Department of Pathophysiology, Nanjing Medical University, Nanjing, China; ^4^School of Life Science, Shanxi University, Taiyuan, China; ^5^Department of Chemistry and Biochemistry, University of Oregon, Eugene, OR, USA

In the past two decades, an increasing number of reports have indicated the remarkable roles of gasotransmitters in biology and medicine. The term gasotransmitter was first coined by Wang in 2002 and further refined in 2012 and 2014 to encompass a group of small gaseous molecules, including nitric oxide (NO), hydrogen sulfide (H_2_S), carbon monoxide (CO), and possibly other gases [1–3]. A gasotransmitter typically has high lipid solubility and can penetrate cell membranes without requiring a specific transporter or receptor. Gasotransmitters are generated endogenously by specific enzymes and can generate various functions at physiologically relevant concentrations by targeting specific cellular and molecular targets. Abnormal generation and metabolism of these gasotransmitters have been extensively demonstrated to be linked to diverse biological processes, such as vascular biology, immune functions, cellular survival, metabolism, longevity, and development and stress resistance.

This specific issue contains both review papers and original research articles that highlight novel discoveries and recent progress in relation to cellular function, molecular mechanisms, and drug targets of gasotransmitters in biology and in medicine as well as the involvement of gasotransmitters in response to environmental stressors in plants. Gasotransmitters have well-defined functions in the vascular system by regulating vascular contraction and dilation since both H_2_S and NO act as endothelial derived hyperpolarizing factors or endothelium-derived relaxing factors [4]. In an original research article, L. Xiao et al. elucidated the protective effect of H_2_S on the endothelium by using a rat two-kidney one-clip hypertensive model. Exogenous administration of H_2_S-releasing donor NaHS lowered blood pressure and improved endothelium dependent contractions. These findings were attributed to the BMP4/ROS/p38 MAPK/COX-2 pathway in the H_2_S-dependent endothelial function, further suggesting the potential therapeutic value of H_2_S in clinical hypertension. In another study, W.-W. Zheng and colleagues demonstrated that NO is involved in high sodium-stimulated activation of epithelial sodium channel (ENaC) in human umbilical vein endothelial cells. High sodium concentrations inhibited endothelial nitric oxide synthase (eNOS) phosphorylation (Ser 1177) levels and NO production, while the specific ENaC blocker, amiloride, reversed this process; therefore NO may contribute to endothelial protection in response to high salt challenge. M. Forte et al. provided a thorough discussion of the possibility of targeting NO with natural derived compounds as a therapeutic strategy for vascular diseases.

In addition to its cardioprotective effects, H_2_S provides potent protection in other systems, including neural system and gastrointestinal endocrine system. A review article by S. Panthi et al. summarized the pathophysiological roles of H_2_S in the central nervous system as well as in peripheral nerve degeneration and regeneration. The authors concluded that a full understanding of H_2_S and its complex interactions with neural units could lead to potential therapeutic strategies that employ H_2_S. D. Wu and colleagues explored the protective roles of H_2_S in obesity-induced kidney injury in mice. They found that H_2_S is able to reduce intrarenal lipid deposits, improve kidney function, and reduce the interstitial injury and fibrosis of the kidney through the reduction of kidney inflammation by downregulating NF-*κ*B expression. These data suggested that H_2_S or its releasing compounds may serve as a potential therapeutic molecule for obesity-induced kidney injury. A review paper by J. Pichette and J. Gagnon further discussed the regulation of H_2_S on glucose metabolism and insulin secretion in both health and disease. Specially, they highlighted the potential roles of H_2_S in the gastrointestinal endocrine system, possibly by direct interaction with the insulin-stimulating incretin hormones (insulinotropic polypeptide and/or glucagon peptide-1).

J. Nevoral et al. summarize recent knowledge on the action of gasotransmitters in maturing oocytes and early embryonic development in various animal species, including sea urchin,* Xenopus*, and mammalian models, pointing to the essential role of gasotransmitters in the beginning of life. They suggested that gasotransmitter regulation of gametogenesis may occur through cysteine residue modification of target proteins, including formation of nitrosothiols and persulfides. Further studies of gasotransmitters on gametogenesis are necessary to further establish the potential for advancement of human assisted reproductive technology and reproduction therapy.

In addition to NO, CO, and H_2_S, sulfur dioxide (SO_2_) has recently been suggested to be a potential gasotransmitter. J. Liu et al. reported investigations into the role of SO_2_ in vascular structural remodeling. SO_2_ may regulate vascular remolding by affecting smooth muscle proliferation and apoptosis, the balance between matrix metalloproteinase and tissue inhibitors of metalloproteinases, oxidative stress, the TGF-*β*1/Smad2/3 pathway, and so forth, all of which are closely related to the pathogenesis of hypertension. The authors also suggested that more clinical data are needed to demonstrate the potential therapeutic target for SO_2_ in cardiovascular diseases.

Apart from the large volume of studies demonstrating their important roles in mammalian systems, the effects of gasotransmitters in plants are now recently being recognized, suggesting the gasotransmitters may act as universal signalling molecules. Z.-J. Ni et al. demonstrated that H_2_S effectively alleviates postharvest senescence of grapes by preventing rachis browning and berry rotting, thus maintaining grape firmness, soluble solids, and titratable acidity during postharvest storage. The protective role of H_2_S in grapes could be attributed to the induction of antioxidant enzymes and attenuation of lipid peroxidation, thereby maintaining the stability of cellular membrane structure.

In a special research paper, S. Brazier et al. reported the functional interactions between BKCa *α*-subunit and Annexin A5 in cell apoptosis. They found that the physical partnership of Annexin A5 and BKCa *α*-channels results in decreased Ca^2+^ sensitivity and removal of the Annexin A5 from the vicinity of the intracellular C-terminal of BKCa *α*-subunit, resulting in augmentation of K^+^ efflux and subsequent apoptosis.

The research on gasotransmitters is quickly expanding and knowledge associated with the potential of gasotransmitters in biology and medicine is rapidly accumulating. It is clear that gasotransmitters play important roles in both health and diseases. Fully understanding the complex molecular mechanisms of gasotransmitters and developing gasotransmitter-related donors and/or inhibitors will be critical for boosting the progress from basic research to clinical or other commercial applications.

## References

[1] Wang R. (2002). Two's company, three's a crowd: can H_2_S be the third endogenous gaseous transmitter?. *The FASEB Journal*.

[2] Wang R. (2012). Physiological implications of hydrogen sulfide: a whiff exploration that blossomed. *Physiological Reviews*.

[3] Wang R. (2014). Gasotransmitters: growing pains and joys. *Trends in Biochemical Sciences*.

[4] Wang R. (2009). Hydrogen sulfide: a new EDRF. *Kidney International*.

